# Integrated Model of Multiple Kernel Learning and Differential Evolution for EUR/USD Trading

**DOI:** 10.1155/2014/914641

**Published:** 2014-07-06

**Authors:** Shangkun Deng, Akito Sakurai

**Affiliations:** Graduate School of Science and Technology, Keio University, 3-14-1 Hiyoshi, Kohoku-ku, Yokohama 223-8522, Japan

## Abstract

Currency trading is an important area for individual investors, government policy decisions, and organization investments. In this study, we propose a hybrid approach referred to as MKL-DE, which combines multiple kernel learning (MKL) with differential evolution (DE) for trading a currency pair. MKL is used to learn a model that predicts changes in the target currency pair, whereas DE is used to generate the buy and sell signals for the target currency pair based on the relative strength index (RSI), while it is also combined with MKL as a trading signal. The new hybrid implementation is applied to EUR/USD trading, which is the most traded foreign exchange (FX) currency pair. MKL is essential for utilizing information from multiple information sources and DE is essential for formulating a trading rule based on a mixture of discrete structures and continuous parameters. Initially, the prediction model optimized by MKL predicts the returns based on a technical indicator called the moving average convergence and divergence. Next, a combined trading signal is optimized by DE using the inputs from the prediction model and technical indicator RSI obtained from multiple timeframes. The experimental results showed that trading using the prediction learned by MKL yielded consistent profits.

## 1. Introduction

The foreign exchange (FX) market is considered to be the largest financial market in the world. In the last few decades, currency trading has received considerable attention from researchers, individual investors, international trade companies, and government organizations. However, there is a problem with predicting directional change in the FX because it is affected by many factors, including financial policy, market mood, or even natural disasters such as earthquakes.

In general, researchers use technical indicators as features of the raw stock prices or FX rates. A technical indicator of stock prices or FX rates is a function that returns a value for given prices over a given length of time in the past. These technical indicators might provide traders with guidance on whether a currency pair is oversold or overbought, or whether a trend will continue or halt. Moving average (MA) [1] is the best-known technical indicator and it is also the basis of many other trend-following or overbought/oversold indicators. The MA is inherently a follower rather than a leader, but it reflects the underlying trend in many cases. Many well-known advanced technical indicators are based on the MA, such as the MACD [2], RSI [3], BIAS ratio [4], and Bollinger Bands [5]. In general, the MACD is used to capture a trend while the RSI, BIAS ratio, and Bollinger Bands are used to provide an early warning of an overbought or oversold currency pair. Traders can follow the trend if it continues but they should also be cautious not to miss overbought or oversold signals related to the target trading stocks or currency pairs.

Previous researchers have used technical indicators such as some MA based methods to identify trends or used technical indicators such as the RSI, William %R, or BIAS ratio to determine whether a target currency pair has been overbought or oversold. For example, Jaruszewicz and Mańdziuk [6] applied technical analysis to predict the Japanese NIKKEI index and so they claimed that the technical indicators are useful in a short time as a day for time horizon. Deng et al. [7] used several technical indicators such as RSI, BIAS ratio, and William %R to generate trading rules by calculating a linear combination of three technical indicators and a stock price change rate predicted value. Wei et al. [8] used several technical indicators such as RSI, MA, and William %R and their values calculated from historical prices were used as conditional features. Chong and Ng [9] predicted the London Stock Exchange based on technical indicators such as the MACD and RSI to generate trading rules, such as “a buy signal is triggered when the RSI crosses the center line (50) from below, while a sell signal is triggered when the RSI crosses the center line (50) from above,” and they found that trading strategy based on RSI or MACD obtained better return than buy-and-hold strategy. Comparing with the previous research of Jaruszewicz and Mańdziuk [6] and Chong and Ng [9], in our proposed method, we used a technical indicator to predict the directional change and used a technical indicator to find overbought/oversold conditions and then to combine a directional change signal with a trade signal from an overbought/oversold indicator, which may provide more reliable trading signal.

In recent years, machine learning techniques have been used increasingly as alternative methods to help investors or researchers forecast directional changes in stock prices or FX rates. The most popular and useful methods are support vector machines (SVMs) and genetic algorithms (GAs). Researchers often apply SVMs to predict directional changes or GAs to generate trading rules based on combinations of trading parameters. For example, Kamruzzaman et al. [10] used a SVM based model to predict FX rates. Shioda et al. [11] used a SVM for monitoring to predict the high volatility of FX rates. Other researchers have used GAs to generate trading rules. For example, Chang Chien and Chen [12] used a GA based model to generate rules for stock trading by mining associative classification rules. Deng and Sakurai [13] used GA to generate trading rules based on a technical indicator for FX trading. Hirabayashi et al. [14] used a GA to generate rules for FX intraday trading by mining features from several technical indicators. Esfahanipour and Mousavi [15] used a GA to generate risk-adjusted rules for trading.

In addition to GAs, differential evolution (DE) was proposed by Storn and Price [16] and it is a population based stochastic search, which functions as an efficient global optimizer in continuous search domains. DE has been applied successfully in various fields. For example, Worasucheep [17] used DE for forecasting the stock exchange index of Thailand. Takahama et al. [18] used DE to optimize neural networks for predicting stock prices. Peralta et al. [19] compared DE and GA for time series prediction and showed that the performance of DE was better than GA if more than 150 generations were generated.

In addition to SVMs, in the last decade, many researchers have used the multiple kernel learning (MKL) [20, 21] to address the problem of selecting suitable kernels for different feature sets. This technique mitigates the risk of erroneous kernel selection to some degree by taking a set of kernels, deriving a weight for each kernel, and making better predictions based on the weighted sum of the kernels. One of the major advantages of MKL is that it can combine different kernels for various input features. Many researchers have applied MKL in their research fields. For example, MKL was used by Joutou and Yanai [22] for food image recognition. Foresti et al. [23] used MK regression for wind speed prediction and their results outperformed those of several conventional methods. Recently, researchers have used MKL for predicting the FX rate, crude oil prices, and stock prices. For example, Deng et al. [24] used MKL to fuse the information from stock time series and social network service for stock price prediction. Deng and Sakurai [25] used MKL for prediction and trading on crude oil markets. Fletcher et al. [26] used MKL for predicting the FX market from the limit order book. Luss and D'Aspremont [27] employed MKL for predicting abnormal returns based on the news using text classification. Yeh et al. [28] used MKL to predict stock prices on the Taiwan stock market and they showed that MKL was better than SVM for evaluating performances. Deng et al. [7] used MKL to predict short-term foreign exchange rate, and the prediction results of MKL based method are much better than conventional methods, in terms of root mean square (RMSE). The difference between the method proposed in Deng et al. [7] and this study is that the proposed method in this study uses one MKL to predict upward trend and uses another MKL for prediction of downward trend, while the method in Deng et al. [7] is used MKL to predict the change rates of FX rate. The reason for using one MKL to predict upward trend and using another MKL to predict downward trend is that our classification is a three-classification problem (upward trend, downward trend, and unknown). In addition, this study uses one technical indicator (RSI) but from three different timeframes, while Deng et al. [7] used three technical indicators but from one timeframe. Deng et al. [7] used multiple technical indicators because of the differences between different technical indicators since they may provide different trading signals, while this research used multiple timeframes of one technical indicator since different timeframes of the same technical indicator may provide different trading signals. In addition to using individual method, several researchers have used hybrid models for trading stocks or FX rate prediction. For example, Huang and Wu [29] used SVM and GA integrated model for predicting a stock index. Huang [30] combined SVM with GA to produce a stock selection model. The better performances of the hybrid SVM-GA model than individual method (SVM or GA), the superiority of DE to GA [19], and superiority of MKL to SVM [22, 26, 28] inspired us to try a new hybrid model which combines MKL and DE. It is logically expected that a MKL-DE will perform better than the previous methods.

In the present study, we use a hybrid method based on MKL and DE for prediction and to generate the trading rules for trading currency rates. In addition, we noticed that some researchers focused on extreme returns or abnormal movements of stock prices. For example, Beneish et al. [31] used contextual fundamental analysis for stock prediction and they focused only on extreme returns, that is, returns above a threshold. Luss and D'Aspremont [27] used MKL and they focused on abnormal movements, which were movements above a threshold. Inspired by their research, in this study, we use MKL to generate signals for upward trends, downward trends, and no trend. The directional change predictor performs learning to predict the direction of price movements. The direction of movement is classified as an upward trend, a downward trend, or a probabilistic fluctuation. Thus, we simply set a threshold for the absolute values of changes, below which we consider the change to be a fluctuation.

In addition to trends, traders also consider the possibility of overbought or oversold conditions for the target currency pair. For example, if a trader predicts an upward trend but the target currency pair is overbought, that is, at a high level, it will be risky to continue following the trend. We could use a technical indicator as a tool to determine the degree to which the FX pair is oversold or overbought, before generating trading actions (buy, sell, or no trade) based on the overbought or oversold signal. In this study, we define the overbought or oversold signals based on a RSI (refer to Section 2.1.3).

**Figure 1:**
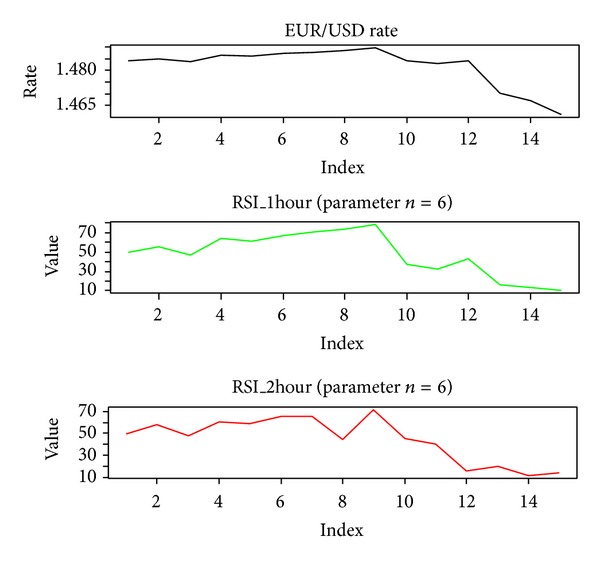
Example showing the relative strength index values from multiple timeframes.

Our trading time horizon is 1 hour, which means that we assess overbought or oversold signals based only on 1-hour time frame data. Clearly, it is possible that the judgment would be different if we made assessments using a longer or shorter timeframe. For example, Figure 1 shows the EUR/USD rate and its RSI values for 1-hour and 2-hour timeframes (i.e., 1-hour RSI and 2-hour RSI values). Note that at the eighth point (10:00:00, May 5, 2011) in Figure 1, the 1-hour RSI value is approximately 73.90, which provides us with a sell signal because the currency pair is overbought, whereas the 2-hour RSI value is approximately 43.98, which tells us that the currency is not overbought. The rate increased further from the eighth to the ninth point (11:00:00, May 5, 2011). In addition, the 1-hour RSI value is approximately 78.32 at the ninth point and the 2-hour RSI value is approximately 71.71, which suggests that both values provide overbought signals so it is highly probable that the rate will decrease from the ninth point onwards. This example shows that if we use the RSI to generate trading rules, we must assess the overbought or oversold conditions not only for the target timeframe, but also for relatively longer and shorter timeframes. For example, the features of the RSI from a relatively shorter timeframe (i.e., 30 minutes in this study) and a relatively longer timeframe (i.e., 2 hours) were used in this study as suitable signals for trading a target currency pair.

In the present study, we use the MACD indicator of two currency pairs as features, rather than only the target currency pair, and the RSI indicator from two different timeframes of the target trading currency pair, rather than the target timeframe.

According to the 2010 Triennial Survey (the share of trading volume), the most heavily traded currency pairs were: EUR/USD 28%, USD/JPY 14%, and GBP/USD 9%. The EUR/USD is the most traded currency pair in the world, so this is used as our target trading currency pair. JPY and GBP are the two most highly exchanged currencies with both USD and EUR, so we also employ GBP/USD and USD/JPY as supplementary information for predicting our target currency pair.

Evaluations of the experimental results should be based on the return-risk ratio as well as the return and the average return, because most investors prefer to obtain stable returns, rather than high returns with high volatility, that is, high risk. Therefore, the Sharpe ratio [32] is used as an evaluation measure to adjust the risk, in addition to the average return.

In summary, this study makes three main innovations, as follows: (1) to predict directional changes of EUR/USD, we set thresholds on the magnitude of the FX rate changes to distinguish upward trend or downward trend from random fluctuations to predict the return, whereas only a few studies employed this process. (2) To generate a trade signal, we fuse information from multiple currency pairs other than only the target currency pair and we combined multiple RSIs from multiple timeframes other than only the target trading timeframe, whereas many previous researchers have considered only the target trading currency pair with a target trading timeframe. (3) The hybrid model combined an upward trend/down ward trend signal with the multiple RSI signal, and the hybrid model yielded greater profits. Proposed model outperformed the baseline and other methods based on the results of return and the return-risk ratio.

The remainder of this paper is organized as follows.  Section 2 describes the background of this research.  Section 3 explains the structure of the proposed method.  Section 4 describes the experimental design.  Section 5 presents the experimental results and provides a discussion.  Section 6 concludes the paper.

## 2. Background

### 2.1. Technical Indicators

Technical indicators are broadly classified into two types: trend indicators and oscillator indicators. The best-known trend indicator is the MA, which is the basis of most other indicators. Next, we introduce the three technical indicators used in this study: MA, MACD as a trend indicator, and RSI as an overbought/oversold indicator.

#### 2.1.1. Simple MA and Exponential MA

The MA is a technique for smoothing out short-term fluctuations, which can be obtained by calculating the mean value of the prices over the past *n*-periods. The MA is used to understand the present trend, which is why it is a so-called trend-following index. There are several types of MA, depending on how past prices are weighted.

The simple MA (SMA) is a simple mean value with identical weights for past prices:

(1) $$\begin{array}{c} {\text{SMA}}_{n}\left(t\right)=\frac{\sum_{k=t-n+1}^{t}P\left(k\right)}{n}, \end{array}$$

where *n* is the period length and *P*(*k*) is the foreign exchange rate or some other value under consideration.

Another type of MA, the exponential MA (EMA), is the mean of the underlying data, which is generally the price of a stock or foreign exchange rate for a given time period *n*, where larger weights are attributed to narrower changes. The difference between the EMA and the SMA is that the EMA is concerned more with the nearest movements, which may have greater effects on future changes than older changes. The EMA is calculated as follows:

(2) $$\begin{array}{c} {\text{EMA}}_{n}\left(t\right)=P\left(t\right)*a+\left(1-a\right)*{\text{EMA}}_{n}\left(t-1\right), \end{array}$$

where EMA_*n*_(*t*) is the EMA of the rate at time *t* and *a* = 2/(*n* + 1), which is commonly used for the *n*-period EMA.

#### 2.1.2. MACD

The MACD is used to predict trends in time series data and it provides two indicators: the MACD value and the MACD signal. In general, the MACD value is the difference between the 12-period and 26-period EMAs, as follows:

(3) $$\begin{array}{c} {\text{MACD}}_{\text{value}}\left(t\right)={\text{EMA}}_{12}\left(t\right)-{\text{EMA}}_{26}\left(t\right). \end{array}$$

The MACD signal is equal to the 9-period EMA of the MACD value, as follows:

(4) $$\begin{array}{c} {\text{MACD}}_{\text{signal}}\left(t\right)={\text{EMA}}_{9}\left({\text{MACD}}_{\text{value}}\left(t\right)\right). \end{array}$$

The MACD parameters (12, 26, and 9) can be adjusted to meet the needs of traders. In our study, we simply use the default MACD parameters given above because they are used widely throughout the world.

#### 2.1.3. RSI

In general, traders use the RSI as a momentum oscillator to compare the magnitude of recent gains with the magnitude of recent losses. If we let *P*(*t*) represent the closing price on day *t*, then we can calculate the gain or loss in period *t* as follows:

(5) $$\begin{array}{c} G_{t}=\{\begin{array}{cc} P\left(t\right)-P\left(t-1\right) & \text{if}\;P\left(t\right)>P\left(t-1\right)\\ 0 & \text{otherwise}, \end{array}\\ L_{t}=\{\begin{array}{cc} P\left(t\right)-P\left(t-1\right) & \text{if}\;P\left(t\right)<P\left(t-1\right)\\ 0 & \text{otherwise}. \end{array} \end{array}$$

Next, the *n*-period average gain (AG(*t*)) is calculated as

(6) $$\begin{array}{c} \text{AG}\left(t\right)=\frac{n-1}{n}\times \text{AG}\left(t-1\right)+\frac{1}{n}\times G_{t}, \end{array}$$

and the *n*-period average loss (*AL*⁡(*t*)) is calculated as

(7) $$\begin{array}{c} \mathrm{AL}\left(t\right)=\frac{n-1}{n}\times \mathrm{AL}\left(t-1\right)+\frac{1}{n}\times L_{t}. \end{array}$$

Thus, the *n*-period RSI at time point *t* is calculated as

(8) $$\begin{array}{c} {\text{RSI}}_{n}\left(t\right)=\frac{\text{AG}\left(t\right)}{\text{AG}\left(t\right)+\mathrm{AL}\left(t\right)}\times 100. \end{array}$$

Traditionally, a RSI value higher than 70 indicates that the currency has been overbought, whereas a value below 30 indicates that the currency pair has been oversold. Thus, the RSI provides alarm signals for investors to close the current position or to open a new position to buy when the currency is oversold and to sell when it is overbought. The parameters used for the overbought and oversold levels can be set up by traders. In the present study, we use DE to optimize the RSI parameter.

### 2.2. SVM and MKL

A SVM is an optimal hyperplane used to separate two classes or a nonlinear separating surface optimized using a nonlinear mapping from the original input space into a high-dimensional feature space to search for an optimally separating hyperplane in the feature space. The latter solves classification problems that cannot be linearly separated in the input space. We designate a hyperplane as optimal if it has a maximal margin, where the margin is the minimal distance from the separating hyperplane to the closest data points, which are called the support vectors.

The concept used to map the data from the original feature space to a high-dimensional feature space is called a kernel method. Finding the optimal hyperplane is formalized as follows:

(9) min⁡ 12||w||2+C∑i=1nζis.t.    yi(〈w·xi〉+b)≥1−ζi,ζi≥0, ∀i=1,2,…,n,

where *w* is the vector of the parameters that define the optimal decision hyperplane 〈*w* · *x*_*i*_〉 + *b* = 0 and *b* represents the bias. (1/2)||*w*||^2^ is considered to be a regularization term, which controls the generalization capacities of the classifier. The second term *C*∑_*i*=1_^*n*^ *ζ*_*i*_ is the empirical risk (error). *C* is sometimes referred to as the soft margin parameter and it determines the tradeoff between the empirical risk and the regularization term. Increasing the value of *C* gives greater importance to empirical risk relative to the regularization term. Positive slack variables *ζ*_*i*_ allow classification errors.

To extend SVM, MKL uses multiple kernels to map the input space to a higher-dimensional feature space by combining different kernels to obtain a better separation function. In MKL, the kernels are combined linearly and the weight of each kernel reflects its importance. The kernels can be different kernels or the same kernels with different parameters. Each kernel in the combination may account for a different feature or a different set of features. The use of multiple kernels can enhance the performance of the model.

Suppose *k*_*m*_  (*m* = 1,…, *M*) are *M* positive definite kernels on the same input space. Finding the optimal decision surface is formalized as follows:

(10) min⁡w,b,ζ 12∑m=1M1dm||Fm||Hm2+C∑i=1Nζi∑i=1nXi2,s.t.   yi(∑m=1M〈Fm,Φm(xi)〉+b)≥1−ζi,ζi≥0, ∀i=1,2,…,n,  ∑m=1Mdm=1, dm≥0,

where Φ is a possibly nonlinear mapping from the input space to a feature space, *F*_*m*_ is the separation function, |||| is a norm, 〈, 〉 is the inner product, *C* is used to control the generalization capacities of the classifier, which is selected by crossvalidation, and *d*_*m*_ are the optimized weights.

In our study, the optimized weights *d*_*m*_ directly represent the ranked relevance of each feature used in the prediction process. We employ MKL to learn the coefficients and parameter of the subkernels. We used the multiple kernel learning toolbox SHOGUN [21] in our experiments.

In our MKL based models, similarity is measured based on the instances of EUR/USD, instances of USD/JPY, and instances of GBP/USD. We construct three similarity matrices for each data source. These three derived similarity matrices are also taken as three subkernels of MKL and the weights of *d*_*m*,EURUSD_, *d*_*m*,GBPUSD_, and *d*_*m*,USDJPY_ are learnt for the subkernels:

(11) k(xi→,xj→)=dm,EURUSDkEURUSD(x→i(1),x→j(1)) +dm,GBPUSDkGBPUSD(x→i(2),x→j(2)) +dm,USDJPYkUSDJPY(x→i(3),x→j(3)),

where $\vec{x_{i}},\;i=1,2,\ldots ,n$, are training samples, *d*_*m*,EURUSD_, *d*_*m*,GBPUSD_, and *d*_*m*,USDJPY_ ≥ 0, and *d*_*m*,EURUSD_ +  *d*_*m*,GBPUSD_ + *d*_*m*,USDJPY_ = 1. *x* ^(1)^ are EUR/USD instances, *x* ^(2)^ are GBP/USD instances, and *x* ^(3)^ are USD/JPY instances. In this study, *k* is the RBF (radial basis function) kernel for SVM and MKL. For other types of information sources or subkernel combinations, similar distance based similarity matrices and kernel functions can be constructed, which are easily imported into our multikernel based learning framework.

### 2.3. DE

The DE method proposed by Storn and Price [16] is a population based stochastic search approach, which can be used as an efficient global optimizer in a continuous search domain. Like other evolutionary algorithms, DE also has a population with the size *N*_*p*_ and *D*-dimensional parameter vectors (*D* is the number of parameters present in an objective function). Two other parameters used in DE are the scaling factor *F* and the crossover rate *C*_*r*_.

#### 2.3.1. Population Structure

The current population, represented by *P*_*x*_, comprises the vectors *x*_*i*_^(*G*)^, which have already been found to be acceptable, either as initial points or based on comparisons with other vectors, as follows:

(12) Px(G)=(xi(G)) i=0,1,…,NP−1,  G=0,1,…,gmax⁡,xi(G)=(xi,j(G)) j=0,1,…,D−1.

After initialization, DE mutates randomly selected vectors to produce an intermediary population *P*_*v*_^(*G*)^ of *N*_*p*_ mutant vectors *V*_*i*_^(*G*)^. Consider

(13) Pv(G)=(Vi(G)) i=0,1,…,NP−1,  G=0,1,…,gmax⁡,Vi(G)=(Vi,j(G)) j=0,1,…,D−1.

Each vector in the current population is recombined with a mutant to produce a trial population *P*_*u*_ of *N*_*p*_ trial vectors *u*_*i*_^(*G*)^. Consider

(14) Pu(G)=(ui(G)) i=0,1,…,NP−1,  G=0,1,…,gmax⁡,ui(G)=(ui,j(G)) j=0,1,…,D−1.

#### 2.3.2. Initialization

Before the population can be initialized, the upper and lower bounds of each parameter must be specified. They can be collected into two *D*-dimensional initialization vectors, *x*_*U*_ and *x*_*L*_. After the initialization bounds have been specified, a random number generator assigns each element of every vector with a value from the prescribed range. For example, the initial value (*G* = 0) of the *j*th parameter of the *i*th vector is

(15) P(0)=xi,j(0)=xj,L+randj[0,1]·(xj,U−xj,L)i=0,1,…,NP−1; j=0,1,…,D−1,

where rand_*j*_[0,1] is a random number, which is generated uniformly between 0 and 1.

#### 2.3.3. Mutation

After initialization, DE mutates and recombines the population to produce a population of *N*_*p*_ trial vectors. A mutant vector is produced according to the following formulation:

(16) Vi,j(G)=xr1,j(G−1)+F·(xr2,j(G−1)−xr3,j(G−1))i=0,1,…,NP−1; j=0,1,…,D−1.

The scale factor *F* is a positive real number, which controls the rate of population evolution. There is no upper limit to *F*, but effective values are seldom greater than 1. *r*1, *r*2, and *r*3 refer to three randomly selected indices from the population.

#### 2.3.4. Crossover

DE also employs uniform crossover. Sometimes referred to as discrete recombination, crossover builds trial vectors from elements that have been copied from two different vectors. In particular, DE crosses each vector with a mutant vector:

(17) $$\begin{array}{c} u_{i,j}^{\left(G\right)}=\{\begin{array}{cc} v_{i,j}^{\left(G\right)} & \text{if}\;\left({\text{rand}}_{i,j}^{\left(G\right)}\leq C_{r}\;\text{or}\;j=j_{\text{rand}}\right)\\ x_{i,j}^{\left(G-1\right)} & \text{otherwise}, \end{array} \end{array}$$

where the crossover probability *C*_*r*_ ∈ [0,1] is a user-defined value, which controls the fraction of elements that are copied from the mutant. To determine the source that contributes, a given uniform crossover compares *C*_*r*_ to a uniform random number rand_*i*,*j*_^(*G*)^ between 0 and 1. If the random number is less than or equal to *C*_*r*_, the trial element is inherited from the mutant *V*_*i*_^(*G*)^; otherwise the element is copied from the vector *x*_*i*_^(*G*−1)^. In addition, the trial element with the randomly selected index *j*_rand_ is taken from the mutant to ensure that the trial vector does not duplicate *x*_*i*_^(*G*)^.

#### 2.3.5. Selection

If the trial vector *u*_*i*_^(*G*)^ has an equal or lower objective function value than that of its target vector *x*_*i*_^(*G*)^, it replaces the target vector in the next generation; otherwise the target retains its place in the population for at least one more generation:

(18) $$\begin{array}{c} x_{i}^{\left(G+1\right)}=\{\begin{array}{cc} u_{i}^{\left(G\right)} & \text{if}\;f\left(u_{i}^{\left(G\right)}\right)\leq f\left(x_{i}^{\left(G\right)}\right)\\ x_{i}^{\left(G\right)} & \text{otherwise}. \end{array} \end{array}$$

#### 2.3.6. Stopping Criteria

After the new population is generated, the processes of mutation, recombination, and selection are repeated until the optimum is obtained, or a user-defined termination criterion, such as the number of generations, is reached at a preset maximum *g*_max⁡_.

### 2.4. Evaluation Measures

In the present study, we performed simulated trading using test samples based on the trading signals generated by MKL prediction and the multiple RSI signal, and we evaluated the return (gain or loss) obtained with the proposed model and other models. In general, a high return is inevitably accompanied by the potential for high risk. Therefore, investors desire a method that decreases risk while not decreasing the profits greatly, which results in a trade-off relationship. The Sharpe ratio, named after William Forsyth Sharpe, is a measure of the excess return per unit of risk in an investment asset or a trading strategy, which is defined as follows:

(19) $$\begin{array}{c} S=\frac{E\left[R-R_{f}\right]}{\sigma }=\frac{E\left[R-R_{f}\right]}{\sqrt{\text{var}\left[R-R_{f}\right]}}, \end{array}$$

where *R* is the asset return, *R*_*f*_ is the return on a benchmark asset (usually a very low risk return such as a three-month US treasury bill), *σ* is the standard deviation of the asset return, and *E*[*R* − *R*_*f*_] is the expected value of the excess of the asset return relative to the benchmark asset return [32]. In our experiments, we used the Sharpe ratio as an evaluation measure to assess the return-risk ratio performance of our proposed method with other methods.

## 3. Proposed Method

### 3.1. Structure of the Proposed Method

**Figure 2:**
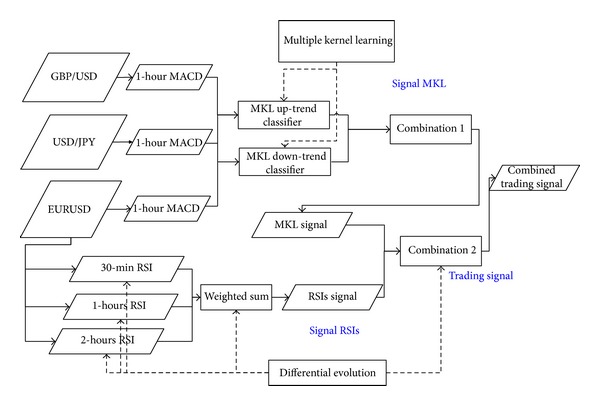
Structure of the proposed method.

Figure 2 shows the structure of the proposed method. First, the proposed method uses a MKL framework to predict directional changes in the currency rate based on the MACD of three currency pairs. The RSI signals are generated using multiple timeframe features of EUR/USD by considering the MKL trading signals. Finally, the MKL signal and RSIs signal are combined to produce a final decision, that is, the trading signal.

The prediction and trading target currency pair in this study is EUR/USD. We selected it as our target due to the fact that the euro and US dollar are the two most traded currencies in the world, representing the world's two largest economies. Therefore, to better predict the changes in EUR/USD is considered to contribute much to the investors and international companies. In addition to EUR/USD data itself, since the two most traded currencies with USD and EUR in FX market are JPY and GBP, USD/JPY and GBP/USD are used for EUR/USD prediction. These three currency pairs share almost 50% of the FX market; other currencies such as AUD (Australian dollars), CAD (Canada dollars), and CHF (Swiss Franc) are also important currencies but since their shares in FX market are relatively small, we did not consider them in the structure of the proposed method.

The trading time interval is selected to be one hour in this study, which is also selected by Hirabayashi et al. [14]. To find overbought/oversold indicator values other than target 1-hour horizon data and to select some reasonable longer and shorter time horizons data are important. Since the trading time interval is one hour, 30-minute and 2-hour time horizon data are considered to be useful. Too high frequency time horizon data (such as minute data) or too low frequency time horizon data (such as daily data) are considered to have small impact if we fix the trading time interval to be one hour.

In this proposed method, we use MKL to predict directional changes and DE to find overbought/oversold information from RSI indicator. Although the predicted directional change can be used for simulated trading, in our preliminary experiments, the accumulated profits based on just the MKL predictions were not good enough (refer to Section 5.1); the same was true for accumulated profits based on using just DE and RSI indicator. Considering that the prediction and the technical indicators might have complementary components, we propose to combine them to get the trading signal. Therefore, we combine MKL and DE in the proposed method.

### 3.2. MKL Input and Output

For MKL, the input features are derived from three different sources: EUR/USD, GBP/USD, and USD/JPY. We transform the rates to MACD signals and values. For each kernel, the inputs are the MACD values and MACD signals for eight consecutive periods, which are shown in Table 1.

Using MKL, we construct two classifiers to output the MKL-up labels and the MKL-down labels (MKL-up refers to an upward trend classifier learned by MKL, while MKL-down refers to a downward trend classifier learned by MKL). We want to predict directional changes in a currency with an insensitive interval, where the changes from −0.1% to 0.1% are not considered upward or downward. Thus, we set two threshold values, that is, 0.1% and −0.1%, which we refer to as the uptrend threshold value and the downtrend value, respectively, to label the training and testing samples. The rules for the MKL-up trend and MKL-down trend classifiers are shown in Table 2.

Based on the predictions of these two MKL classifiers, we obtain a combined MKL signal based on the rules, which are shown in Table 3. The combined MKL trading signal is one of the inputs for DE that needs to be combined with the multiple RSI signal.

### 3.3. Combined Trading Signal Based on the Combined MKL and Multiple RSI Signals

The multiple RSI signal value Value_RSIs_ is the combined value of three timeframe RSI values:

(20) $$\begin{array}{c} {\text{Value}}_{\text{RSIs}}=\overset{3}{\underset{i=1}{\sum }}w_{i}e_{i}, \end{array}$$

where *w*_*i*_ are the weights of the three RSIs and *e*_*i*_ is the value of the RSI indicator. Note that the value of the RSI indicator is expressed as a ratio and we use RSI/100 from (8). The weights *w*_*i*_ of each RSI are learned by DE.

We compare the RSI values in (20) with the buy/sell threshold to determine the multiple RSI signal. The signal and the condition that need to be satisfied before the signal can be issued are shown in Table 4.

Signal_trading_ is a signal used for making decisions based on both the combined MKL signal and the multiple RSI signal.  Table 5 shows how the combined MKL and multiple RSI signal are combined to obtain the trading signal. If we decide to take a position (buy or sell), the position is retained for 1 hour; that is, we check the conditions every hour. If the trading signal (buy or sell) is the same as that 1 hour before, we do not trade and we wait for 1 hour. The data we use are 1-hour EUR/USD (we used 30 min data to calculate the 30 min RSI value, and 1-hour data to calculate the 1-hour RSI value and the 2-hour RSI value).

### 3.4. DE Parameter Design

The DE parameter vectors shown in Table 6 are used to construct the multiple RSI signals. The representations of the parameter vectors are as follows.

1. The first three groups represent the parameters for each RSI (three RSIs in total). The values range from 3 to 10 (integer type).
2. Numbers 4 to 5 are used to decide the times to buy, sell, and close positions. The values range from 0 to 2 (floating point number type).
3. Numbers 6 to 8 are the weights used to linearly combine signals, which are described in (20) in Section 3.3. The values range from 0 to 1 (floating point number type).

The population size is set to 200 and the maximum number of generations is set to 200 during the DE training step. The accumulated return obtained in the training step is selected as the objective function.

## 4. Experiment Design

The exchange rates used in this study were obtained from ICAP. The ICAP data was used in our previous study [13] for trading on EUR/USD. The ICAP data use the GMT +1 hour time zone (GMT +2 hour in summer) and they do cover the exchange rate in weekend. A list of best offers, best bids, and dealt prices for every second are comprised in the ICAP data. We transformed them into 30 min and 1-hour timeframes. We used exchange rate data for three currency pairs from ICAP data: EUR/USD, GBP/USD, and USD/JPY. We separate the overall data into three datasets and each dataset covered the period from January 3 to December 30 in each year, with a total of about 6200 observations (hourly data). The three datasets used for training and testing are shown in Table 7.

The data include the “open, high, low, and close” rates during each time interval (30 min and 1 hour). The data were divided into three disjoint datasets that covered consecutive periods, the details of which are shown in Table 8. Next, we divided each dataset into a training period and a testing period. The MKL training period covered 3000 observations (around 6 months) and the testing period covered 3000 observations (around 6 months). The MKL-DE training step covered 1500 trading hours and the MKL-DE testing step covered 1500 trading hours. Details of the length of each period are shown in Table 8.

Foreign exchange market is often and suddenly affected by economic events such as a bank rate decision or even unpredictable affair such as a big earthquake. Therefore, in a trading in the experiments, our initial investment is *A* US dollars. For each transaction (long or short), we fix the trading amount to be *A*/2 US dollars with a trading leverage ratio of 2 to 1. That is, although we did margin transaction, the trading in our experiments is conducted with very low leverage (or with a very high margin level), which ensures the safety of our transaction order even though there is a big shock in FX market.

Table 9 shows a list of the methods tested, including baseline methods, proposed methods, and intermediate methods. “Buy and hold” and “sell and hold” were selected as baseline methods because they are simple and well known, while they are the best methods for obtaining zero profit on average if the market is efficient and stationary. The trading rule they used was to buy or sell at the start of the testing period and to close the position at the end of the testing period. The other methods used for comparison comprising the simplest methods and our proposed methods. SVM-s used a kernelized linear model for exchange rates where the inputs were the exchange rates of only one currency pair with SVM as a learning method. SVM-m was the same as SVM-s but it utilized the features of three currency pairs. MKL-m was the same as SVM-m but the model was a multiple kernelized linear model that uses MKL. MKL-m-t and MKL-m-t-DE were the same as MKL-m but the prediction was changed to a three-classification problem from a two-classification problem. The trading rule used by SVM-s, SVM-m, and MKL-m was to buy a currency pair when the prediction was positive, to sell when negative, and “no trade” when the prediction was 0. The trading rule for MKL-m-t was based on Signal_MKL_. The trading rule used by MKL-m-t-DE, our proposed method, was based on Signal_trading_ where the parameters were optimized using MKL and DE (see Table 5). DE-only was based on Signal_RSIs_; that is, it relied only on multiple RSI signals. The DE algorithm includes random numbers, so we conducted 10 experiments with different seeds for MKL-m-t-DE and DE-only. In the list of methods tested, since GA based method are well-known methods in the previous literatures [12–14], GA-s and GA-m which are implemented by Deng and Sakurai [13] are considered as benchmark methods, and we conducted 10 experiments with different seeds for GA-s and GA-m. “Buy and hold” and “sell and hold” are well-known baseline methods which are also used as baseline methods by Chong and Ng [9]; SVM-s, SVM-m, MKL-m, MKL-m-t, DE-only, and MKL-m-t-DE are implemented by us.

## 5. Experimental Results and Discussion

### 5.1. Returns with the Three Datasets

Table 10 shows the returns with the methods tested, where the returns were measured in proportion to the initial investment (the entries in the first three columns for MKL-m-t-DE, DE-only, GA-s, and GA-m are the average returns from 10 independent experiments with their standard deviations). First, we found that during the testing period (three months) for each dataset, our proposed method yielded good average returns (about 6.73%, 4.71%, and 3.52%). In addition, our proposed method obtained the best average return (4.98%) among all the methods tested.

Next, we focused on the baseline methods: “buy and hold” and “sell and hold.” We found that “buy and hold” yielded losses with all three testing datasets while “sell and hold” yielded better returns than the other methods except MKL-m-t-DE during the three testing periods. The “sell and hold” strategy yielded profits during the testing periods because EUR had declined against USD due to the European sovereign debt crisis [33], which occurred in the Eurozone after a big rise in EUR against USD from 2005 until the first half of 2008. We could not forecast the decline or surge before this period, so we could not decide whether “buy and hold” was better than “sell and hold” and we could not conclude that these two naïve strategies performed well.

In addition, we compared the results with SVM-s and SVM-m. Table 10 shows that these SVM based methods yielded losses during all three testing periods. SVM-m used more information (the features of three FX pairs) than SVM-s (the features of EUR/USD only) in dataset 2 (2009), but the return with SVM-m (−3.2%) was not better than that with SVM-s (−2.2%).

Moreover, we compared the results of proposed method with that of GA-s and GA-m. Table 10 shows that GA-s yielded positive return on average during 2008, while yielded losses on average during 2009 and 2010. GA-m yielded positive return in 2008 and 2010, but it yielded losses on average during 2009 and the average return of three data sets is about −0.004, which is much worse than the results of our proposed method. In addition, the average return results of GA-m for the three data sets are better than those of GA-s, which agrees with the conclusion in Deng and Sakurai [13] that the return results improved when using information of RSI indicator from multiple timeframes.

Based on the average returns, we found that MKL-m-t performed better than MKL-m, which indicated that the returns were improved by neglecting small predicted changes such as fluctuations in the MKL-m method. DE-only used DE alone to generate the trading rules based on multiple RSI values, but it yielded losses on average. MKL-m-t-DE performed the best of the four methods (MKL-m, MKL-m-t, MKL-m-t-DE, and DE-only), which indicates that the integration of multiple RSI signals could improve the trading performance.

### 5.2. Sharpe Ratios

In addition to the returns, the Sharpe ratio was used to evaluate the performance of our proposed method and other methods. We used the one-year treasury rate as the risk-free asset to calculate the Sharpe ratio. The one-year treasury rate ranged from 1.7% to 4.3% between 2008 and 2010. Next, we calculated the average risk-free returns from 2008 to 2010 and the average risk-free return for each testing period (three months in each year) was about 0.75%.  Table 11 shows the average returns, standard deviations, and Sharpe ratios with each method (for the methods “MKL-m-t-DE” and “DE-only,” “average return” results are the averages of all the returns obtained from 10 experiments for all the testing periods with all the datasets, while the “standard deviation” is the standard deviation of these returns).

A higher Sharpe ratio indicates a higher return or lower volatility. From Table 11, we found that for the methods “GA-s,” “GA-m,” “buy and hold,” “SVM-s,” “SVM-m,” “MKL-m,” and “DE-only,” their Sharpe ratio values are negative, which indicates that their average return is less than the free-risk asset. There are three methods that obtained positive Sharpe ratio value: “sell and hold,” “MKL-m-t,” and our proposed method “MKL-m-t-DE.” It is clear that our proposed method had a significantly higher Sharpe ratio (2.6111) than the other two methods during the testing periods. The Sharpe ratio results indicate that the proposed method is the best method when evaluated by return-risk ratio.

## 6. Conclusion and Future Work

In this study, we developed a hybrid method based on MKL and DE for EUR/USD trading. In the first step of our approach, we used MKL to predict the directional change in the currency rate (with an insensitive interval) to provide a combined MKL signal. In the second step, DE combined the combined MKL signal with the multiple RSI signal to generate a trading signal. The experimental results showed that MKL-m-t yielded profits with the three testing datasets (about 1.38% on average), while integration of the multiple RSI signal improved the trading profits (about 4.98% on average). In addition, the proposed method yielded the best Sharpe ratio (about 2.61) compared with all the models tested, which indicates that our proposed method outperformed other methods in terms of the return-risk ratio, as well as the returns.

However, there are still some unaddressed questions and some research directions for future work. For example, how to find the best insensitive internal (−0.1% to 0.1% in this study) is still an open question in this study: a too large insensitive interval could decrease the number trading times too much so that the trading profit also decreases, while a too small insensitive interval cannot filter the unknown movements well the trading profit decreases. For future work, one may combine MKL with GA to use GA to search the best parameters for insensitive interval in MKL automatically in order to solve the unaddressed problems. In addition, other than RSI, some other famous overbought/oversold indicators, such as BIAS and William %R, could be also implemented to improve the trading ability.

## Figures and Tables

**Table 1:** Features for each kernel.

No.	Feature
1	MACD-value at time *t*
2	MACD-signal at time *t*
3	MACD-value at time (*t* − 1)
4	MACD-signal at time (*t* − 1)
5	MACD-value at time (*t* − 2)
6	MACD-signal at time (*t* − 2)
7	MACD-value at time (*t* − 3)
8	MACD-signal at time (*t* − 3)
9	MACD-value at time (*t* − 4)
10	MACD-signal at time (*t* − 4)
11	MACD-value at time (*t* − 5)
12	MACD-signal at time (*t* − 5)
13	MACD-value at time (*t* − 6)
14	MACD-signal at time (*t* − 6)
15	MACD-value at time (*t* − 7)
16	MACD-signal at time (*t* − 7)

**Table 2:** Output labels for MKL up-trend and down-trend classifiers.

MKL classifier	MKL-trend signal	Conditions
MKL-up trend	MKL-up = +1	If the actual change rate is greater than the upward trend threshold value
	MKL-up = −1	If the actual change rate is less than the upward trend threshold value

MKL-down trend	MKL-down = +1	If the actual change rate is less than the downward trend threshold value
	MKL-down = −1	If the actual change rate is greater than the downward trend threshold value

**Table 3:** Conditions for issuing the MKL signal.

No	Combined MKL signal (Signal_MKL_)	Conditions
1	No trade	MKL-up = 1 and MKL-down = 1
2	No trade	MKL-up = −1 and MKL-down = −1
3	Buy	MKL-up = 1 and MKL-down = −1
4	Sell	MKL-up = −1 and MKL-down = 1

**Table 4:** Conditions that need to be satisfied before issuing the RSI signal.

No	Multiple RSI signal (Signal_RSIs_)	Conditions
1	Buy	Value_RSIs_ < buy threshold
2	Sell	Value_RSIs_ > sell threshold
3	No trade	otherwise

**Table 5:** Conditions that need to be satisfied before issuing the trading signal.

Trading signal (Signal_trading_)	Conditions	
	Combined MKL signal (Signal_MKL_)	Multiple RSI signal (Signal_RSIs_)
Buy	Buy	No trade
Sell	Sell	No trade
No trade	No trade	No trade
Sell	Any (buy, sell, or no trade)	Sell
Buy	Any (buy, sell, or no trade)	Buy

**Table 6:** DE parameter vector design.

No	Value	Description
1	3 to 10	parameter for 1-hour RSI
2	3 to 10	parameter for 2-hour RSI
3	3 to 10	parameter for 30-min RSI
4	0 to 2	buy threshold
5	0 to 2	sell threshold
6	0 to 1	weight value for 1-hour RSI
7	0 to 1	weight value for 2-hour RSI
8	0 to 1	weight value for 30-min RSI

**Table 7:** Three datasets used for training and testing.

Dataset	MKL training	MKL testing	MKL-DE training	MKL-DE testing
Dataset 1 (2008)	Jan. to Jun.	Jul. to Dec.	Jul. to Sep.	Oct. to Dec.
Dataset 2 (2009)	Jan. to Jun.	Jul. to Dec.	Jul. to Sep.	Oct. to Dec.
Dataset 3 (2010)	Jan. to Jun.	Jul. to Dec.	Jul. to Sep.	Oct. to Dec.

**Table 8:** Trading and testing periods for MKL and DE.

Period	Process	Length of period
1	MKL learning	3000 trading hours (around 6 months)
2	MKL testing (prediction)	3000 trading hours (around 6 months)
2-1	MKL-DE training	1500 trading hours (around 3 months)
2-2	MKL-DE testing (trading)	1500 trading hours (around 3 months)

**Table 9:** List of the methods tested.

Method	Description
GA-s	Trade based on the trading rules optimized by GA, with one RSI input
GA-m	Trade based on the trading rules optimization by GA, with three RSI input
Buy and hold	Buy and hold until the end point of a period
Sell and hold	Sell and hold until the end point of a period
SVM-s	Trade based on SVM prediction, with one FX pair input
SVM-m	Trade based on SVM prediction, with three FX pairs input
MKL-m	Trade based on MKL prediction, with three FX pairs input
MKL-m-t	Trade based on Signal_MKL_
DE-only	Trade based on Signal_RSIs_ (parameters are optimized by DE)
MKL-m-t-DE	Trade based on Signal_trading_

**Table 10:** Returns with the methods tested (The numbers right to ± is the standard deviation).

Method	Dataset 1 (2008)	Dataset 2 (2009)	Dataset 3 (2010)	Average returns
GA-s	0.0068 ± 0.0230	−0.0454 ± 0.0143	−0.0284 ± 0.0569	−0.0223
GA-m	0.0098 ± 0.0991	−0.0326 ± 0.0286	0.0087 ± 0.0241	−0.0046
Buy and hold	−0.0510	−0.0426	−0.0229	−0.0388
Sell and hold	0.0510	0.0426	0.0229	0.0388
SVM-s	−0.2039	−0.0225	−0.0559	−0.0941
SVM-m	−0.0397	−0.0324	−0.0299	−0.0340
MKL-m	−0.1932	−0.0103	0.0479	−0.0518
MKL-m-t	0.0216	0.0150	0.0048	0.0138
DE-only	0.0035 ± 0.0991	−0.0318 ± 0.0541	0.0082 ± 0.0131	−0.0201
MKL-m-t-DE	0.0673 ± 0.0343	0.0471 ± 0.0362	0.0352 ± 0.0215	0.0498

**Table 11:** Sharpe ratios for the baseline, benchmark, and proposed methods.

Method	Average return	Standard deviation	Sharpe ratio
GA-s	−0.0223	0.0242	−0.5025
GA-m	−0.0046	0.0266	−1.1177
Buy and Hold	−0.0388	0.0144	−3.2152
Sell and Hold	0.0388	0.0144	2.1736
SVM-s	−0.0941	0.0965	−1.0528
SVM-m	−0.0340	0.0050	−8.3000
MKL-m	−0.0518	0.1258	−0.4713
MKL-m-t	0.0138	0.0084	0.7500
DE-only	−0.0201	0.0219	−1.2602
MKL-m-t-DE	0.0498	0.0162	2.6111
